# Altered Maternal Circulating and Placental microRNA-369-5p and microRNA-671-3p Expression in Intrahepatic Cholestasis of Pregnancy: Comparison of ICSI and Spontaneous Conception

**DOI:** 10.3390/ijms27188019

**Published:** 2026-09-09

**Authors:** Sibel Bulgurcuoglu Kuran, Meryem Hocaoglu, Selin Demirer, Esra Kaynak, Ilayda Loçlar Karaalp, Elvan Yagimli Ozturk, Ayse Altun, Ibrahim Sogut, Canan Hurdag, Abdulkadir Turgut, Evrim Komurcu Bayrak

**Affiliations:** 1 Department of Obstetrics and Gynecology, Reproductive Endocrinology and Infertility, IVF Unit, Istanbul Medical Faculty, Istanbul University, 34093 Istanbul, Turkey; 2 Department of Obstetrics and Gynecology, Goztepe Prof Dr. Suleyman Yalcin City Hospital Affiliated to Istanbul Medeniyet University, 34722 Istanbul, Turkey; dr.meryemtaskiran@gmail.com (M.H.);; 3 Department of Genetics, Aziz Sancar Institute of Experimental Medicine, Istanbul University, 34093 Istanbul, Turkey; 4 Department of Biochemistry, Faculty of Medicine, Demiroglu Bilim University, 34394 Istanbul, Turkey; 5 Department of Histology and Embryology, Faculty of Medicine, Demiroglu Bilim University, 34394 Istanbul, Turkey

**Keywords:** intrahepatic cholestasis of pregnancy, intracytoplasmic sperm injection, microRNA-369-5p, microRNA-671-3p, leukocytes, plasma, placenta

## Abstract

To investigate microRNA-369-5p (miR-369-5p) and miR-671-3p expression in maternal peripheral blood leukocytes, plasma, and placental tissue in women with intrahepatic cholestasis of pregnancy (ICP) undergoing intracytoplasmic sperm injection (ICSI), compared with spontaneous conception. In this exploratory cohort study, microRNA-369-5p (miR-369-5p) and microRNA-671-3p (miR-671-3p) expression levels were analyzed using real-time quantitative PCR in maternal peripheral blood leukocytes, plasma, and placental tissue in four groups: (1) women with uncomplicated pregnancies conceived via intracytoplasmic sperm injection (non-ICP-ICSI, *n* = 7), (2) women with intrahepatic cholestasis of pregnancy conceived via intracytoplasmic sperm injection (ICP-ICSI, *n* = 7), (3) women with intrahepatic cholestasis of pregnancy who conceived spontaneously (ICP-SC, *n* = 7), and (4) women with uncomplicated pregnancies conceived spontaneously (non-ICP-SC, *n* = 7). Relative expression levels were calculated using the RQ method. Leukocyte miR-369-5p expression was significantly upregulated in ICP-ICSI and non-ICP-ICSI groups compared with ICP-SC and non-ICP-SC groups (*p* = 0.0001). Plasma miR-671-3p expression levels were significantly decreased in ICSI groups compared with spontaneous conception groups (*p* = 0.029). No significant differences were observed in placental expression levels of miR-369-5p and miR-671-3p among the four groups (*p* > 0.05). In subgroup analysis, leukocyte miR-369-5p and miR-671-3p expression levels were significantly higher in the ICP-ICSI group compared with the ICP-SC group (*p* < 0.05). Serum fasting bile acid levels showed a significant positive correlation with placental miR-369-5p expression (r = 0.467; *p* = 0.012) and a negative correlation with plasma miR-369-5p expression (r = −0.413; *p* = 0.029). Our findings show compartment-specific differences in miR-369-5p and miR-671-3p expression across leukocytes and plasma in this exploratory cohort. These preliminary observations do not establish causality and require validation in larger, independent cohorts.

## 1. Introduction

Intrahepatic cholestasis of pregnancy (ICP) is a reversible pregnancy-specific liver disorder characterized by pruritus and elevated serum bile acid (SBA) levels, typically occurring in the late second or third trimester [1]. The incidence of ICP varies widely between <1% and 27.6% worldwide due to ethnic, geographical, and seasonal differences [2,3]. ICP is associated with adverse fetal and neonatal outcomes, including intrauterine fetal demise (IUFD), meconium-stained amniotic fluid, preterm birth, intrapartum fetal distress, and hypoxic–ischemic events [4].

Recent evidence suggests that ICP is more frequently observed in pregnancies conceived through assisted reproductive technology (ART) compared with spontaneous conceptions, in both singleton and multiple gestations [5]. Altered hormonal milieu, including elevated umbilical cord estradiol and progesterone levels, together with abnormal implantation and gestational microenvironment in in vitro fertilization (IVF) pregnancies, may contribute to the increased susceptibility to ICP in these cases [6,7]. In addition, a widely accepted hypothesis proposes that increased estrogen levels may impair bile acid transport in genetically predisposed individuals, leading to reduced biliary excretion and elevated serum bile acids, liver enzymes, and bilirubin levels [8].

MicroRNAs (miRNAs) are endogenous, single-stranded, non-coding RNA molecules of approximately 22 nucleotides that regulate gene expression at the post-transcriptional level [9]. The miR-369-5p gene is located within the Chromosome 14 miRNA cluster (C14MC), which plays an important role in placental development [10]. miR-369-5p has been shown to be downregulated in differentiated cells and may regulate metabolic splicing factors and cellular reprogramming processes [11,12]. Furthermore, miR-369-5p has been reported to inhibit proliferation, migration, and invasion of hepatocellular carcinoma cells by targeting HOXA13 [12]. Recently, hsa-miR-369-5p expression was found to be significantly upregulated in urine samples from patients with ICP compared with normal pregnancies [13].

miR-671, encoded on chromosome 7q36.1, has been implicated in the pathogenesis of several disorders, including different cancer types, atherosclerosis, ischemic stroke, liver fibrosis, and osteoarthritis, suggesting a potential role in metabolic regulation [14]. Clinically, miR-671-3p has been proposed as a potential non-invasive biomarker for prenatal screening of placenta accreta spectrum disorders [15]. In addition, miR-671-3p expression has been reported to be significantly upregulated in urine samples from ICP patients compared with healthy pregnancies [13].

Although several studies have investigated miRNA profiles in ICP, no previous study has evaluated miR-369-5p and miR-671-3p expression in maternal leukocytes, plasma, and placental tissue in ICP pregnancies conceived via ART compared with spontaneous conceptions. Therefore, the aim of this study was to investigate the expression of miR-369-5p and miR-671-3p in maternal peripheral blood leukocytes, plasma, and placenta in women with ICP undergoing intracytoplasmic sperm injection (ICSI) compared with spontaneous conception.

## 2. Results

### 2.1. Maternal Characteristics and Perinatal Outcomes of the Study Population

There was no significant difference in age, BMI at the time of delivery, and smoking status among the groups (*p* > 0.05). When the age at ICP diagnosis was compared between ICP-ICSI (35.10 ± 3.12) and ICP-SC groups (34.01 ± 1.97), no significant difference was observed (*p* = 0.45). No significant difference was seen in serum fasting bile acid level between ICP-ICSI (21.94 ± 13.56) and ICP-SC groups (15.87 ± 5.62) (*p* = 0.125). As expected, total and conjugated bilirubin, ALP, AST, and ALT serum levels were significantly higher in the ICP-ICSI and ICP-SC groups compared to the non-ICP-SC group (*p* < 0.05). Maternal demographic and clinical characteristics of the study groups were described in Table 1. GA at delivery, mode of delivery, umbilical pH, NICU admission, the presence of fetal distress, and meconium-stained fluid were not significantly different among the four groups (*p* > 0.05). The perinatal outcomes of the study population were shown in Table 2.

### 2.2. Expression Profile of miR-369-5p and miR-671-3p in Maternal Blood Leukocytes, Plasma, and Placenta Among the Study Groups

Blood leukocyte miR-369-5p expression levels were significantly upregulated in the ICP-ICSI (RQ value = 11.46 ± 2.34) and the non-ICP-ICSI groups (RQ value = 9.28 ± 2.50), compared with the ICP-SC (RQ value = 2.29 ± 2.89) and the non-ICP-SC groups (RQ value = 0.00 ± 1.129) (*p* = 0.0001) (Table 3). There was a significant decrease in the expression levels of plasma miR-369-5p in the ICP-ICSI (RQ value = −1.55 ± 2.23) and the ICP-SC groups (RQ value = −1.39 ± 0.89), compared to non-ICP-ICSI (RQ value = 1.64 ± 2.79) and non-ICP-SC groups (RQ value = 0.00 ± 2.44) (*p* = 0.042). On the other hand, no significant difference was seen in the placental expression levels of miR-369-5p among the four groups (*p* = 0.150). Our study showed increased expression levels of blood leukocyte miR-671-3p in ICP-ICSI (RQ value = 10.68 ± 2.45) and non-ICP-ICSI groups (RQ value = 7.32 ± 2.01), compared to ICP-SC (RQ value = 2.12 ± 1.45) and non-ICP-SC groups (RQ value = 0.00 ± 0.87) (*p* = 0.0001). A significant decrease in the expression levels of plasma miR-671-3p in the ICP-ICSI (RQ value = −1.42 ± 1.65) and non-ICP-ICSI groups (RQ value = −2.73 ± 1.95), compared to ICP-SC (RQ value = −0.23 ± 0.37) and non-ICP-SC groups (RQ value = 0.00 ± 2.38) (*p* = 0.029). There was no significant difference in the placental expression levels of miR-671-3p among the four groups (*p* = 0.247). Table 3 indicated the expression levels of miR-369-5p and miR-671-3p in maternal blood leukocytes, plasma, and placenta among the study groups.

**Figure 1:**
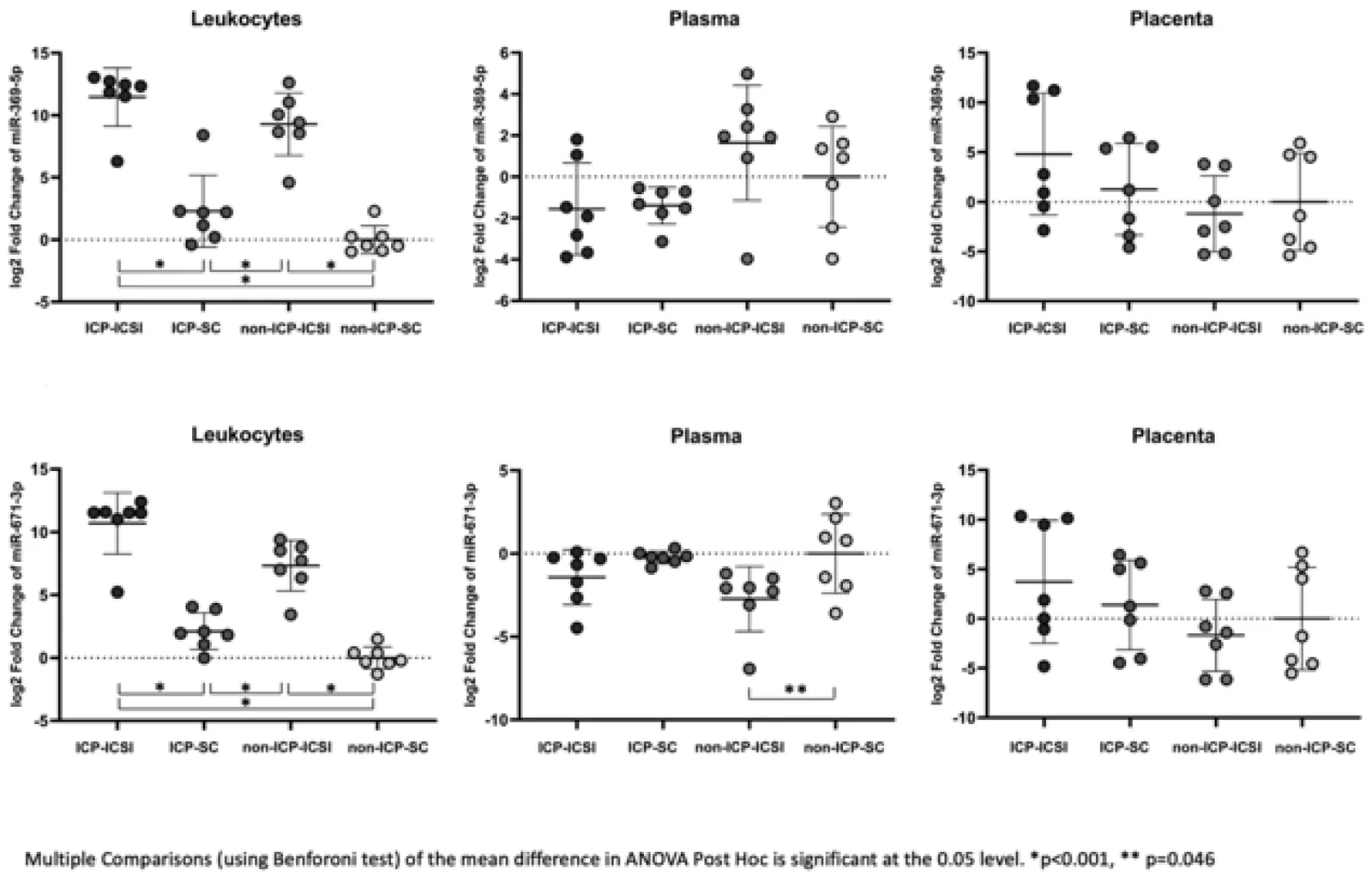
The pairwise comparisons of microRNA-369-5p and microRNA-671-3p expression levels among the study groups.

Leukocyte miR-369-5p and miR-671-3p expression levels in the ICP-ICSI (RQ value = 11.46 ± 2.34 and 10.68 ± 2.45, respectively) exhibited significant upregulation compared with the expression levels of leukocyte miR-369-5p and miR-671-3p in the non-ICP-SC group (RQ value = 0.00 ± 1.12 and 0.00 ± 0.87, respectively) (*p* < 0.001) (Figure 1). Similarly, leukocyte miR-369-5p and miR-671-3p expression levels in the ICP-ICSI group (RQ value = 11.46 ± 2.34 and 10.68 ± 2.45, respectively) were significantly increased compared to those in the ICP-SC group (RQ value = 2.29 ± 2.89 and 2.12 ± 1.45, respectively) (*p* < 0.001). Moreover, miR-369-5p and miR-671-3p expression levels of leukocytes in the non-ICP-ICSI group (RQ value = 9.28 ± 2.50 and 7.32 ± 2.01, respectively) were found to be upregulated compared to those in the non-ICP-SC group (RQ value = 0.00 ± 1.12 and 0.00 ± 0.87, respectively) (*p* < 0.001). On the other hand, miR-671-3p expression levels of plasma in the non-ICP-ICSI group (RQ value = −2.73 ± 1.95) were significantly downregulated compared to those in the non-ICP-SC group (RQ value = 0.00 ± 2.38, respectively) (*p* = 0.046). Figure 1 showed the pairwise comparisons of miR-369-5p and miR-671-3p expression levels among the study groups.

### 2.3. Correlation Analysis Between Clinical Parameters and the Expression Levels of miR-369-5p and miR-671-3p Within the Study Population

The correlation coefficient for leukocyte, plasma, and placental miR-369-5p expression levels and clinical variables using Pearson’s correlation were given in Table 4, Table 5 and Table 6. A significantly negative correlation was observed between placental miR-369-5p and plasma miR-369-5p expression levels (r = −0.545; *p* = 0.003) (Table 4). There was a negative correlation between leukocyte miR-671-3p and plasma miR-671-3p expression levels (r = −0.401; *p* = 0.034). Serum fasting bile acid levels were significantly positively correlated with placenta miR-369-5p expression levels (r = 0.467; *p* = 0.012) and negatively correlated with plasma miR-369-5p expression levels (r = −0.413; *p* = 0.029) (Table 5). Serum total bilirubin levels were significantly negatively correlated with plasma miR-369-5p expression levels (r = −0.50; *p* = 0.007) (Table 5). Moreover, a significantly positive correlation was observed in serum fasting bile acid levels and placental miR-671 expression levels (r = 0.430; *p* = 0.022) (Table 6). Birth weight was found to be significantly positively correlated with plasma miR-671 expression levels (r = 0.486; *p* = 0.009) (Table 6).

## 3. Discussion

MiR-369-5p and miR-671-3p have been implicated in the etiopathogenesis of adipogenic differentiation of mesenchymal stromal cells, diabetes, impaired insulin secretion, cardiac fibrosis, and hepatocellular carcinoma [12,16,17,18,19,20]. Previous studies have also demonstrated downregulation of miR-369-5p in differentiated cells, suggesting a role in regulating metabolic splicing factors and cellular reprogramming processes [11]. As a member of the maternally imprinted C14MC cluster, which plays an important role in human embryonic development, miR-369 has been associated with adverse pregnancy outcomes, including severe preeclampsia, preterm birth, and small-for-gestational-age (SGA) infants [10,21]. Östling et al. reported decreased miR-369-5p expression in placentas from SGA infants; however, this association was not significant after adjustment for confounders [21]. In our study, no significant difference in SGA incidence was observed among the four groups.

In contrast, Roxenlund et al. reported downregulation of miR-3679-5p in SGA neonates exposed to low maternal gestational weight gain, suggesting a link between nutritional status, insulin/IGF-1 signaling, and placental growth regulation [22].

Ma et al. reported upregulated urinary miR-369-5p expression in ICP patients compared with healthy pregnancies and demonstrated diagnostic utility when combined with other miRNAs, yielding high sensitivity and specificity [13].

In our study, we found a significant decrease in plasma miR-369-5p expression levels in the ICP-ICSI and the ICP-SC groups compared to the non-ICP-ICSI and non-ICP-SC groups, whereas no significant difference was seen in the placental expression levels of miR-369-5p among the four groups. Moreover, serum fasting bile acid levels were significantly positively correlated with placental miR-369-5p expression levels and negatively correlated with plasma miR369-5p expression levels. Serum total bilirubin levels were significantly negatively correlated with plasma miR-369-5p expression levels. Given our study findings, miR-369-5p expression levels in plasma seem to be associated with ICP diagnosis. The link between miR-3695p and ICP pathogenesis is more likely due to the fact that ICP is considered to be related to an abnormal metabolic profile, including glucose intolerance and dyslipidemia, as growing evidence suggested [23].

Interestingly, leukocyte miR-369-5p expression was significantly upregulated in ICSI pregnancies, regardless of ICP status, suggesting a stronger association with ART procedures. This may reflect immune or inflammatory activation related to assisted reproductive technologies. Indeed, miR-369-5p has been linked to TGF-β1 signaling pathways, further supporting its potential role in immune regulation [24]. Notably, leukocyte miR expression did not parallel plasma or placental expression, indicating compartment-specific regulation of miRNAs.

miR-671-3p has been reported to be dysregulated in several malignancies, including glioma, colorectal cancer, and hepatocellular carcinoma, and may participate in metabolic pathways [14]. It has also been proposed as a potential biomarker in placenta accreta spectrum disorders [15] and osteoarthritis [18]. Ma et al. demonstrated increased urinary miR-671-3p expression in ICP patients, supporting its potential diagnostic value [13].

The results of our research revealed up-regulated expression levels of leucocyte miR-671-3p in ICP-ICSI and non-ICP-ICSI groups, compared to ICP-SC and non-ICP-SC groups. Leukocyte miR-671-3p expression levels in the ICP-ICSI exhibited significant upregulation compared with the expression levels of leukocyte miR-671-3p in the nonICP-SC group. Leucocyte miR-671-3p expression levels in the ICP-ICSI group were significantly increased compared to those in the ICP-SC group. Moreover, miR-671-3p expression levels of leucocytes in the non-ICP-ICSI group were found to be upregulated compared to those in the non-ICP-SC group. On the other hand, we showed a significant decrease in the expression levels of plasma miR-671-3p in the ICP-ICSI and non-ICPICSI groups, compared to ICP-SC and non-ICP-SC groups. MiR-671-3p expression levels of plasma in the non-ICP-ICSI group were significantly downregulated compared to those in the non-ICP-SC group.

Moreover, we did not observe significant difference in the placental expression levels of miR-6713p among the four groups, whereas a significantly positive correlation was observed in serum fasting bile acid levels and placental miR-671 expression levels. Based on our findings, miR-671 may play a role in the pathogenesis of ICP and could serve as a potential biomarker for the disease. Given that the pathogenesis of ICP is closely associated with abnormal metabolic profiles, including glucose intolerance and dyslipidemia, it is plausible that miR-671 is involved in both metabolic regulation and the development of ICP [18,23]. Additionally, it may be speculated that pregnancies achieved through ICSI could exacerbate the condition and significantly influence miR expression. The interplay between ICSI and ICP may stem from the molecular and hormonal alterations associated with ART. Even though we did not observe significant difference in the placental expression levels of miR-671-3p among the groups, ART procedures are known to be associated with altered placental miR expression, which may contribute to impaired placental angiogenesis [25]. In fact, we observe a significantly positive correlation in serum fasting bile acid levels and placental miR-671 expression levels. We found a negative correlation between leucocyte miR-6713p and plasma miR-671-3p expression. The variation in plasma and leukocyte miR concentrations may indicate individual biological differences rather than pre-analytical factors are known to influence circulating miRNA measurements [26,27,28].

The absence of significant differences in placental miR-369-5p and miR-6713p expression, despite significant alterations in leukocyte and plasma levels, may indicate compartment-specific regulation of these miRNAs. This finding suggests that changes detected in maternal circulating compartments may not necessarily parallel placental expression. However, the mechanisms underlying these differences cannot be determined from the present data and require further investigation. Although placental samples were collected immediately after delivery from a standardized region 4–5 cm from the umbilical cord insertion, potential effects of spatial placental heterogeneity and sampling timing on miRNA expression cannot be completely excluded.

ICP is associated with altered bile acid homeostasis and inflammatory processes. Elevated bile acid levels may contribute to pro-inflammatory signaling, providing a possible biological context for the distinct miRNA expression patterns observed in maternal leukocytes and plasma [29]. In another inflammatory disease model, miR-369-5p has been associated with inflammatory responses [30]. However, the present study does not establish that increased leukocyte expression represents cellular accumulation or that reduced plasma levels result from immune-mediated clearance. These findings may instead reflect compartment-specific regulation and should be considered hypothesis-generating.

From a functional perspective, Ma et al. [13] reported altered miR-369-5p and miR-671-3p expression in ICP, supporting their potential relevance to this condition. Tao et al. [19] demonstrated that miR-369-5p targets DNMT3A and is involved in regulation of the Patched1 signaling pathway, whereas bioinformatic analyses by Xiong et al. [31] linked miR-671-3p target genes to pathways including Wnt signaling. However, these findings derive from different biological and disease contexts, and their relevance to ICP and ICSI pregnancies remains uncertain. Therefore, these potential mechanisms should be considered hypothesis-generating and require further functional and bioinformatic validation.

Importantly, the present study is the first to evaluate miR-369-5p and miR-671-3p expression simultaneously in leukocytes, plasma, and placenta in ICP pregnancies conceived via ICSI compared with spontaneous conception. A strength of this study is the inclusion of well-characterized groups supported by power analysis. However, the relatively small sample size, with seven participants in each group, represents an important limitation of this study and may reduce the precision and robustness of the statistical estimates, particularly for correlation analyses. Therefore, the present findings should be interpreted as exploratory and require validation in larger, independent cohorts. The study also did not include functional experiments and therefore cannot establish the mechanisms underlying the compartment-specific expression patterns. Detailed indications for cesarean delivery were not systematically available, limiting interpretation of the relatively high cesarean delivery rate observed in the study groups. Given the exploratory nature of the correlation analyses, no correction for multiple testing was applied; therefore, the possibility of type I error due to multiple comparisons cannot be excluded, and these findings should be interpreted cautiously and considered hypothesis-generating. Future studies should investigate miRNA expression in different ART protocols, including fresh IVF, ICSI, and IUI cycles, to better clarify the role of assisted reproduction in ICP pathophysiology. In addition, the observed differences in miRNA expression cannot be attributed solely to the ICSI procedure, as ART-related factors such as the underlying cause of infertility, hormonal stimulation, embryo cryopreservation, and maternal characteristics may also influence miRNA expression. Furthermore, all ICSI pregnancies in this study resulted from frozen embryo transfer cycles using the same protocol; therefore, these findings should not be generalized to all ART pregnancies. Hemolysis was not quantitatively assessed using a spectrophotometric or miRNA-based method, which should be considered a methodological limitation of the circulating miRNA analysis. In addition, maternal blood samples were obtained at a single time point near delivery; therefore, the present study cannot characterize longitudinal changes in miRNA expression or determine whether the observed leukocyte and plasma patterns develop progressively during pregnancy.

## 4. Material and Methods

### 4.1. Study Subjects

This prospective cohort study was conducted between July 2019 and March 2022 in the antenatal clinic of Goztepe Prof. Dr. Suleyman Yalcin City Hospital affiliated to Istanbul Medeniyet University. A total of 28 singleton Caucasian pregnant women were enrolled. Participants were classified into four groups: (a) normal pregnant women who conceived spontaneously (non-ICP-SC group, *n* = 7), (b) women with ICP who conceived spontaneously (ICP-SC group, *n* = 7), (c) normal pregnant women undergoing ICSI (non-ICP-ICSI group, *n* = 7), and (d) women with ICP undergoing ICSI (ICP-ICSI group, *n* = 7).

ICP was diagnosed based on the following criteria: (1) generalized pruritus during pregnancy without associated skin disease or rash, (2) elevated total serum bile acid (TSBA) concentrations ≥10 μmol/L, (3) elevated serum aspartate aminotransferase (AST) and/or alanine aminotransferase (ALT) levels > 40 U/L, (4) normal ultrasonographic findings of the liver and gallbladder, and (5) negative serologic tests for hepatitis A, B, and C [32].

Women with preeclampsia, gestational diabetes mellitus, known or suspected liver or biliary tract disease, intrauterine fetal demise, fetal chromosomal or structural abnormalities, and multiple pregnancies were excluded. In addition, pregnancies conceived using donor oocytes and ICSI protocols other than the gonadotropin-releasing hormone (GnRH) antagonist protocol commonly used in Turkey were excluded. All ICSI pregnancies included in the study were achieved through frozen–thawed embryo transfer (FET) cycles.

Demographic and clinical characteristics of all participants were recorded, including age, gravidity, parity, pre-pregnancy body mass index (BMI), BMI at delivery, smoking status, history of ICP, gestational age at ICP diagnosis, ursodeoxycholic acid (UDCA) treatment, antenatal corticosteroid use, white blood cell count, hemoglobin, hematocrit, platelet count, serum fasting bile acid, liver function tests [AST (IU/L), ALT (IU/L), gamma-glutamyl transferase (GGT) (IU/L), lactate dehydrogenase (LDH) (IU/L)], total and direct bilirubin, alkaline phosphatase (ALP), glucose levels, gestational age at delivery, mode of delivery, neonatal birth weight, fetal sex, small-for-gestational-age (SGA) status, umbilical cord pH, 5-min Apgar score, fetal distress, meconium-stained amniotic fluid (MSAF), and neonatal intensive care unit (NICU) admission.

Women diagnosed with ICP received UDCA treatment (10–15 mg/kg/day) after fasting venous blood samples were collected. Antenatal corticosteroids were administered as part of standard care to promote fetal lung maturation (one or a maximum of two doses of 12 mg betamethasone intramuscularly, 24 h apart). Gestational age was determined based on the reliable last menstrual period or first-trimester ultrasound measurement of crown–rump length.

### 4.2. Blood Sample and Placental Tissue Collection and RNA Isolation

Peripheral venous blood samples were obtained from the antecubital veins of the volunteers (*n* = 28) participating in the study into a 10 mL EDTA tube just before the delivery. Following collection during a two-hour period, each blood sample was treated with 2 volumes of Gey’s solution (155 mMNH4Cl, 10 mM KHCO3 in DEPC treated distilled water) to lyse the red blood cells (twenty minutes at 4 °C). Placental tissue samples were obtained immediately after delivery, following either cesarean section or vaginal delivery, from a standardized site 4–5 cm from the umbilical cord insertion using forceps and a scalpel. Fresh placental tissue samples were cut into smaller pieces and immediately kept frozen in liquid nitrogen and stored at −80 °C. Leukocyte and 100 mg placenta tissue samples were homogenized in TRIzol Reagent (Invitrogen, cat. no. 15596026), and total RNA was isolated from peripheral leukocytes, as described in detail in our previous study [33]. RNA was isolated from the plasma samples according to the manufacturer’s instructions of Quick-cfRNA Serum/Plasma Kit (Zymo Research, Irvine, CA, USA, cat. no. R1059). The quality and quantity of extracted RNA samples were measured using a NanoDrop 1000 spectrophotometer (Thermo Scientific, Wilmington, DE, USA). Then, the RNA samples were stored at −80 °C. Plasma samples were processed promptly after blood collection, and no visible hemolysis was observed. Samples with A260/A280 ratios between 1.8 and 2.2 were included in the RT-qPCR analyses.

### 4.3. Quantification of miR-369-5p and miR-671-3p

cDNA synthesis was performed from RNAs with a concentration of 10 ng/µL using the Qiagen miScript II RT (Qiagen, Hilden, Germany; cat. no. 218161) synthesis kit. The expression levels of miR-369-5p and miR-671-3p were measured according to the manufacturer’s instructions using the miScript SYBR Green PCR Kit (Qiagen, Hilden, Germany; cat. no. 218073). The hsa-miR-369-5p (cat. no. MS00006860) and hsa-miR-671-3p (Qiagen, Hilden, Germany; cat. no. MS00037639) target-specific miScript Primer Assays and miScript Universal Primer (Qiagen, Hilden, Germany; cat. no. 218300) were used for miR-369-5p and miR-671-3p. The hsa-RNU6 (Qiagen, Hilden, Germany; cat. no. MS00033740) assay for tissue and leukocyte samples and the hsa-miR-423-3p (Qiagen, Hilden, Germany; cat. no. MS00004179) assay for plasma samples were used as endogenous controls [34]. The amplification was performed in a LightCycler 480 System (Roche, San Francisco, CA, USA) in triplicate in 96-well plates. The reactions were initiated in a 96-well plate at 95 °C for 15 min with a ramping rate of 4.4 °C/s, followed by 45 cycles of 94 °C for 15 s, 55 °C for 30 s, 70 °C for 30 s with a ramping rate of 1.0 °C/s. The amplification curves were analyzed using the LC480 software (LightCycler® 480 Software, Version 1.5 (Roche Diagnostics, Mannheim, Germany), both for determination of cross point (Cp) values (by the second derivative method) and for melting curve analysis. The 2^−ΔΔCt^ method, in which ΔΔCT = (CTmiRNA − CTnormalization) case group − (CTmiRNA − CTnormalization) control group consisting of normal pregnant women who conceived spontaneously (*n* = 7) was used to calculate the relative quantitation values (fold change—FC) of samples. The stability of RNU6 and hsa-miR-423-3p across the study groups was evaluated, and no statistically significant differences in raw Ct values were observed between groups (*p* > 0.05). Standard curves were generated to determine PCR amplification efficiencies, which ranged from 90% to 105%. A Ct cut-off value of 35 was applied, and no amplification was considered above this threshold.

### 4.4. Statistical Analysis

The normality of all quantitative variables was assessed using the Shapiro–Wilk test in combination with visual inspection of histograms and Q–Q plots. Continuous variables exhibiting a skewed distribution, specifically raw miRNA expression levels, were subjected to log_2_ transformation to improve normality and variance homogeneity prior to analysis.

Statistical analyses were performed using SPSS 14.0 (SPSS Inc., Chicago, IL, USA). While categorical variables were compared using chi-square (χ2), continuous variables were compared using Student’s *t*-test or one-way analysis of variance (ANOVA), as appropriate. The relative expression levels of candidate miRNAs were measured based on the threshold cycle (CT) value. All normalized data were log2-transformed prior to performing ANOVA and Bonferroni’s post hoc test and expressed as fold change. The fold change (relative quantitation level (RQ)) values of miRNAs in leukocytes, plasma, and placenta samples were compared using ANOVA among the four groups, and the results were presented as mean ± standard deviation (S.D.). In addition, pairwise multiple comparisons were performed by using ANOVA and Bonferroni test to compare fold changes values of miRNAs among the study groups. A *p*-value < 0.05 and log2 (fold change) ≠ 0 suggested differentially expressed miRNAs between the four groups [35]. The miRNAs with log2 (fold change) > 0 were characterized as upregulated miRNAs, although miRNAs with log2 (fold change) < 0 were characterized as downregulated miRNAs [35]. A *p* value less than 0.05 was considered as statistically significant. In the whole study cohort (*n* = 28), correlations between miRNA expression levels and individual clinical parameters were evaluated using Pearson’s correlation coefficient. Bonferroni correction was not applied to these correlation analyses. For multiple comparisons of miRNA expression levels among the four study groups following ANOVA, Bonferroni’s post hoc test was applied. Correlation analyses were considered exploratory, and no adjustment for multiple testing was applied.

### 4.5. Sample Size and Power Calculation

G*Power software (version 3.1) was used to perform an a priori power analysis [36]. Based on a one-way ANOVA, an effect size (Cohen’s f) of 0.75 and a significance level of α = 0.05, a total sample size of 28 participants (7 participants in each of the four groups) was estimated to provide a statistical power of 88%.

## 5. Conclusions

To our knowledge, this study is the first to compare maternal leukocyte, plasma, and placental miR-369-5p and miR-671-3p expression profiles in intrahepatic cholestasis of pregnancy conceived through ICSI and spontaneous conception. The observed leukocyte and plasma expression patterns were associated with conception mode and ICP status in this exploratory cohort, whereas no statistically significant differences were detected in placental expression. These findings are preliminary and do not establish a causal effect of ICSI or an immune-mediated clearance mechanism. Validation in larger, independent cohorts is required to confirm these associations and to explore their potential translational relevance.

## Figures and Tables

**Table 1:** Maternal demographic and clinical characteristics of the study population.

Variables	ICP-ICSI Group (*n* = 7) Mean ± SD	ICP-SC Group (*n* = 7) Mean ± SD	Non-ICP-ICSI Group (*n* = 7) Mean ± SD	Non-ICP-SC Group (*n* = 7) Mean ± SD	*p* Value
**Age, year**	34.7 ± 6.34	27.14 ± 6.86	30.14 ± 6.46	30.14 ± 7.15	0.23
**Gravidity, *n* (%)**					0.164
**1**	5 (71.4)	5 (71.4)	4 (57.1)	1 (14.3)	
**2**	1 (14.3)	1 (14.3)	2 (28.6)	0 (0)	
**≥3**	1 (14.3)	1 (14.3)	1 (14.3)	5 (71.4)	
**Parity, *n* (%)**	1 (14.3)	4 (57.1)	1 (14.3)	5 (71.4)	0.054
**Primiparous, *n* (%)**	3 (42.9)	2 (28.6)	4 (57.1)	1 (14.3)	0.375
**Pre-pregnancy BMI, kg/m^2^**	21.71 ± 3.40	21.14 ± 3.6	23.71 ± 4.0	26.14 ± 4.56	0.1
**BMI at the time of delivery**	25.71 ± 3.81	27.42 ± 3.15	26.85 ± 4.98	29.85 ± 2.41	0.22
**Current smoking, *n* (%)**	0 (0)	2 (28.6)	0 (0)	1 (14.3)	0.25
**Previous history of ICP, *n* (%)**	0 (0)	4 (57.1)	0 (0)	0 (0)	**0.003**
**GA at diagnosis, weeks**	35.10 ± 3.12	34.01 ± 1.97	-	-	0.45
**Use of UDCA, *n* (%)**	7 (100)	7 (100)	0 (0)	0 (0)	**0.0001**
**Use of antenatal steroid, *n* (%)**	2 (28.6)	3 (42.9)	3 (42.9)	0 (0)	0.241
**Serum fasting bile acid, μmol/L**	21.94 ± 13.56	15.87 ± 5.62	-	-	0.125
**Hemoglobin, g/dL**	11.92 ± 1.13	11.52 ± 0.91	11.64 ± 1.88	11.75 ± 1.59	0.96
**Hematocrit, %**	35.32 ± 3.51	34.70 ± 1.92	34.85 ± 5.39	35.42 ± 5.02	0.98
**White blood cell count, ×10^3^/μL**	11.42 ± 3.16	9.97 ± 2.70	9.97 ± 2.70	11.01 ± 1.86	0.76
**Platelet count, ×10^3^/μL**	259.71 ± 92.05	219.14 ± 76.30	249.57 ± 79.71	213.14 ± 90.07	0.68
**Glucose, mg/dL**	78.85 ± 7.92	80.00 ± 9.93	91.28 ± 12.49	82.28 ± 12.95	0.16
**Total bilirubin (μmol/L)**	086 ± 0.21	0.68 ± 0.19	0.32 ± 0.05	0.44 ± 0.21	**0.0001**
**Conjugated bilirubin (μmol/L)**	0.46 ± 0.12	0.35 ± 0.16	0.14 ± 0.03	0.20 ± 0.07	**0.0001**
**ALP (U/L)**	233.85 ± 95.66	209.14 ± 39.83	121.85 ± 30.38	154.85 ± 61.82	**0.01**
**AST, IU/L**	82.14 ± 37.01	61.85 ± 34.9	22.57 ± 8.46	22.14 ± 9.26	**0.0001**
**ALT, IU/L**	146.57 ± 112.97	97.57 ± 70.20	18.57 ± 17.21	13.42 ± 5.53	**0.002**
**LDH (U/I)**	221.285 ± 47.59	204.00 ± 72.64	190.14 ± 19.03	288.71 ± 88.55	0.34
**GGT (IU/L)**	20.00 ± 16.43	28.85 ± 27.75	11.71 ± 8.9	11.00 ± 7.54	0.20

Abbreviations: ICP, intrahepatic cholestasis of pregnancy; ICSI, intracytoplasmic sperm injection; SC, spontaneous conception; SD, standard deviation; BMI, body mass index; GA, gestational age; UDCA, ursodeoxycholic acid; ALP, alkaline phosphatase; AST, aspartate aminotransferase; ALT, alanine aminotransferase; LDH, lactate de-hydrogenase; GGT, gamma-glutamyl transferase. Values are number (%) or mean ± SD. Categorical variables were analyzed using the chi-square (χ^2^) test. Continuous variables were evaluated using analysis of variance (ANOVA). Bold values denote statistical significance at the *p* < 0.05 level.

**Table 2:** Perinatal outcomes of the study subjects.

Variables	ICP-ICSI Group (*n* = 7) Mean ± SD	ICP-SC Group (*n* = 7) Mean ± SD	Non-ICP-ICSI Group (*n* = 7) Mean ± SD	Non-ICP-SC Group (*n* = 7) Mean ± SD	*p* Value
GA at delivery, week	36.05 ± 2.48	36.80 ± 0.37	35.58 ± 3.54	38.75 ± 0.77	0.06
Mode of delivery, *n* (%)					0.903
Vaginal	1 (14.3)	2 (28.6)	2 (28.6)	2 (28.6)	
Cesarean section	6 (85.7)	5 (71.4)	5 (71.4)	5 (71.4)	
Birth weight, grams	2708.57 ± 532.41	3115.00 ± 591.79	2690.71 ± 745.36	3475.00 ± 354.81	0.05
Fetal sex, female *n* (%)	4 (57.1)	4 (57.1)	6 (85.7)	3 (42.9)	0.416
Umbilical pH	7.34 ± 0.04	7.32 ± 0.06	7.31 ± 0.03	7.32 ± 0.05	0.85
5-min Apgar score, *n* (%)					0.329
6	1 (14.3)	-	2 (28.6)	-	
7	1 (14.3)	-	-	-	
8	-	1 (14.3)	-	-	
9	5 (71.4)	6 (85.7)	4 (57.1)	7 (100)	
10	-	-	1 (14.3)	-	
Fetal distress, *n* (%)	0 (0)	0 (0)	0 (0)	0 (0)	-
Meconium-stained fluid, *n* (%)	1 (14.3)	0 (0)	0 (0)	0 (0)	0.375
SGA, *n* (%)	2 (28.6)	1 (14.3)	2 (28.6)	0 (0)	0.444
NICU admission (*n*) (%)	1 (14.3)	3 (42.9)	2 (28.6)	1 (14.3)	0.553

Abbreviations: ICP, intrahepatic cholestasis of pregnancy; ICSI, intracytoplasmic sperm injection; SC, spontaneous conception; SD, standard deviation; GA, gestational age; SGA, small for gestational age; NICU, Neonatal Intensive Care Unit. Values are number (%) or mean ± SD. Categorical variables were analyzed using the chi-square (χ^2^) test. Continuous variables were evaluated using analysis of variance (ANOVA). *p*-value < 0.05 is considered statistically significant.

**Table 3:** The expression levels of microRNA-369-5p and microRNA-671-3p in maternal blood leukocytes, plasma, and placenta among the study groups.

Log2 Fold-Change in miRNAs	Samples	ICP-ICSI Group (*n* = 7) Mean ± SD	ICP-SC Group (*n* = 7) Mean ± SD	Non-ICP-ICSI Group (*n* = 7) Mean ± SD	Non-ICP-SC Group (*n* = 7) Mean ± SD	*p* Value
**microRNA-369-5p**	**Leukocytes**	11.46 ± 2.34	2.29 ± 2.89	9.28 ± 2.50	0.00 ± 1.12	**0.0001**
	**Plasma**	−1.55 ± 2.23	−1.39 ± 0.89	1.64 ± 2.79	0.00 ± 2.44	**0.042**
	**Placenta**	4.80 ± 6.12	1.26 ± 4.60	−1.21 ± 3.82	0.00 ± 4.90	0.150
**microRNA-671-3p**	**Leukocytes**	10.68 ± 2.45	2.12 ± 1.45	7.32 ± 2.01	0.00 ± 0.87	**0.0001**
	**Plasma**	−1.42 ± 1.65	−0.23 ± 0.37	−2.73 ± 1.95	0.00 ± 2.38	**0.029**
	**Placenta**	3.71 ± 6.21	1.38 ± 4.52	−1.68 ± 3.64	0.00 ± 5.18	0.247

Abbreviations: ICP, intrahepatic cholestasis of pregnancy; ICSI, intracytoplasmic sperm injection; SC, spontaneous conception; SD, standard deviation; Values are number (%) or mean ± SD. Log2 fold-change values were evaluated using analysis of variance (ANOVA). Bold values denote statistical significance at the *p* < 0.05 level.

**Table 4:** Correlation coefficients for leukocyte, plasma, and placental microRNA-369-5p and miRNA-671-3p expression levels using Pearson’s correlation.

	**microRNA-369-5p RQ**								
**log2 Fold-Change in miRNAs**	**Leukocyte microRNA-369-5p RQ**			**Placenta microRNA-369-5p RQ**			**Plasma microRNA-369-5p RQ**		
	**Pearson Correlation**	**Sig. (2-Tailed)**	***N***	**Pearson Correlation**	**Sig. (2-Tailed)**	***N***	**Pearson Correlation**	**Sig. (2-Tailed)**	***N***
**Leukocyte microRNA-369-5p RQ**	1	-	28	0.233	0.233	28	-0.127	0.521	28
**Placenta microRNA-369-5p RQ**	0.233	0.233	28	1	-	28	−0.545 **	**0.003**	28
**Plasma microRNA-369-5p RQ**	−0.127	0.521	28	−0.545 **	**0.003**	28	1	-	28
	**microRNA-671-3p**								
	**Leukocyte microRNA-671-3p RQ**			**Placenta microRNA-671-3p RQ**			**Plasma microRNA-671-3p RQ**		
	**Pearson Correlation**	**Sig. (2-Tailed)**	***N***	**Pearson Correlation**	**Sig. (2-Tailed)**	***N***	**Pearson Correlation**	**Sig. (2-Tailed)**	***N***
**Leukocyte microRNA-671-3p RQ**	1		28	0.271	0.163	28	−0.401 *	**0.034**	28
**Placenta microRNA-671-3p RQ**	0.271	0.163	28	1	-	28	−0.196	0.316	28
**Plasma microRNA-671-3p RQ**	−0.401 *	**0.034**	28	−0.196	0.31	28	1	-	28

Abbreviations: RQ: relative quantitation; bold values denote statistical significance at the *p* < 0.05 level. ** Correlation is significant at the 0.01 level (2-tailed). * Correlation is significant at the 0.05 level (2-tailed).

**Table 5:** Correlation coefficients for leukocyte, plasma, and placental microRNA-369-5p expression levels and clinical variables using Pearson’s correlation.

Variables	Leukocyte microRNA-369-5p RQ			Placenta microRNA-369-5p RQ			Plasma microRNA-369-5p RQ		
	Pearson Correlation	Sig. (2-Tailed)	*N*	Pearson Correlation	Sig. (2-Tailed)	*N*	Pearson Correlation	Sig. (2-Tailed)	*N*
**Hemoglobin, g/dL**	0.312	0.106	28	0.21	0.283	28	−0.287	0.138	28
**Hematocrit, %**	0.257	0.186	28	0.096	0.625	28	−0.263	0.176	28
**White blood cell count, ×10^3^/μL**	0.169	0.389	28	−0.043	0.829	28	0.047	0.811	28
**Platelet count, ×10^3^/μL**	0.231	0.238	28	0.143	0.467	28	−0.233	0.234	28
**Serum fasting bile acid, μmol/L**	0.241	0.216	28	0.467 *	**0.012**	28	−0.413 *	**0.029**	28
**AST, IU/L**	0.209	0.285	28	0.195	0.321	28	−0.28	0.149	28
**ALT, IU/L**	0.164	0.405	28	0.104	0.6	28	−0.125	0.527	28
**LDH, IU/L**	−0.257	0.186	28	0.055	0.78	28	−0.132	0.505	28
**GGT, IU/L**	−0.033	0.867	28	0.348	0.07	28	−0.168	0.393	28
**Glucose, mg/dL**	0.115	0.559	28	-0.298	0.123	28	0.243	0.213	28
**Total bilirubin, μmol/L**	0.22	0.26	28	0.372	0.051	28	−0.500 **	** 0.007 **	28
**Conjugated bilirubin, μmol/L**	0.196	0.317	28	0.355	0.064	28	−0.357	0.062	28
**ALP, U/L**	0.089	0.651	28	0.328	0.089	28	−0.337	0.08	28
**Pre-pregnancy BMI, kg/m^2^**	−0.022	0.911	28	0.021	0.917	28	0.288	0.138	28
**BMI at the time of delivery**	−0.36	0.06	28	−0.063	0.752	28	0.066	0.739	28
**Umbilical pH**	−0.018	0.928	28	0.213	0.276	28	−0.15	0.446	28
**GA at diagnosis, week**	0.408	0.148	28	0.257	0.374	28	−0.395	0.162	28
**GA at delivery, week**	−0.192	0.328	28	0.232	0.234	28	−0.26	0.182	28
**Birth weight,** **grams**	−0.36	0.06	28	−0.063	0.752	28	0.066	0.739	28

Abbreviations: BMI, body mass index; GA, gestational age; ALP, alkaline phosphatase; AST, aspartate aminotransferase; ALT, alanine aminotransferase; LDH, lactate dehydrogenase; GGT, gamma-glutamyl transferase. ** Correlation is significant at the 0.01 level (2-tailed). * Correlation is significant at the 0.05 level (2-tailed).

**Table 6:** Correlation coefficients for leukocyte plasma, and placental miRNA-671-3p expression levels and clinical variables using Pearson’s correlation.

Variables	Leukocyte microRNA-671-3p RQ			Placenta microRNA-671-3p RQ			Plasma microRNA-671-3p RQ		
	Pearson Correlation	Sig. (2-Tailed)	*N*	Pearson Correlation	Sig. (2-Tailed)	*N*	Pearson Correlation	Sig. (2-Tailed)	*N*
**Hemoglobin, g/dL**	0.31	0.108	28	0.207	0.291	28	−0.082	0.678	28
**Hematocrit, %**	0.258	0.185	28	0.102	0.604	28	−0.063	0.751	28
**White blood cell count, ×10^3^/μL**	0.189	0.335	28	−0.041	0.838	28	−0.035	0.858	28
**Platelet count, ×10^3^/μL**	0.248	0.203	28	0.111	0.573	28	−0.314	0.104	28
**Serum fasting bile acid, μmol/L**	0.329	0.087	28	0.430 *	**0.022**	28	0.06	0.762	28
**AST, IU/L**	0.294	0.13	28	0.147	0.457	28	0.039	0.843	28
**ALT, IU/L**	0.246	0.208	28	0.049	0.806	28	0.085	0.666	28
**LDH, IU/L**	−0.234	0.232	28	0.087	0.658	28	0.047	0.813	28
**GGT, IU/L**	0.017	0.931	28	0.345	0.072	28	0.002	0.993	28
**Glucose, mg/dL**	0.02	0.918	28	−0.341	0.075	28	−0.121	0.54	28
**Total bilirubin μmol/L**	0.332	0.085	28	0.355	0.064	28	0.003	0.987	28
**Conjugated bilirubin, μmol/L**	0.301	0.119	28	0.346	0.072	28	0.172	0.38	28
**ALP, IU/L**	0.189	0.336	28	0.349	0.068	28	0.069	0.728	28
**Pre-pregnancy BMI, kg/m^2^**	−0.075	0.706	28	0.061	0.758	28	0.237	0.225	28
**BMI at the time of delivery**	−0.213	0.277	28	−0.052	0.792	28	0.346	0.072	28
**Umbilical pH**	−0.006	0.974	28	0.214	0.275	28	−0.197	0.316	28
**GA at diagnosis, week**	0.521	0.056	28	0.292	0.312	28	−0.271	0.348	28
**GA at delivery, week**	−0.155	0.432	28	0.292	0.132	28	0.2	0.307	28
**Birth weight,** **grams**	−0.301	0.12	28	0.032	0.872	28	0.486 **	** 0.009 **	28

Abbreviations: BMI, body mass index; GA, gestational age; ALP, alkaline phosphatase; AST, aspartate aminotransferase; ALT, alanine aminotransferase; LDH, lactate dehydrogenase; GGT, gamma-glutamyl transferase. ** Correlation is significant at the 0.01 level (2-tailed). * Correlation is significant at the 0.05 level (2-tailed).

## Data Availability

Data Availability Statement: The data presented in this study are available from the corresponding author upon reasonable request.
